# Depression and anxiety among pregnant mothers in the initial stage of the Coronavirus Disease (COVID-19) pandemic in the southwest of Iran

**DOI:** 10.1186/s12978-021-01167-y

**Published:** 2021-06-04

**Authors:** Najmeh Maharlouei, Pedram Keshavarz, Niloufar Salemi, Kamran B. Lankarani

**Affiliations:** 1 Health Policy Research Center, Institute of Health, Shiraz University of Medical Sciences, Shiraz, Iran; 2 Department of Radiology, Medical Imaging Research Center, Shiraz University of Medical Sciences, Shiraz, Iran; 3 Radiology Department Office, Namazi Hospital, Namazi Square, Shiraz, Iran

**Keywords:** COVID-19, Pregnancy, Depression, Anxiety, Mental disorders, DASS-21

## Abstract

**‌Background:**

Women are at a higher risk for depression progression, especially during pregnancy. The current study purposed to investigate depression, anxiety, and stress levels of pregnant mothers in the initial stage of the COVID-19 infection in the southwest of Iran.

**Methods:**

This cross-sectional study was conducted during March and April, 2020, in Shiraz, Iran. Pregnant mothers registered in maternity clinics affiliated with Shiraz University of Medical Sciences were included. An online self-administered checklist was used. It included socio-demographic, obstetric and medical histories, and the short form of the Depression Anxiety Stress Scales (DASS-21) to evaluate depression, anxiety, and stress. A *p*-value < 0.05 was considered significant.

**Results:**

In total, 540 pregnant mothers answered the questionnaire. 83.5% had no comorbidity. Abnormal depression scores were significantly higher in those who had no insurance (OR = 2.5) and in those with poor self-rated health (SRH) (OR = 27.8). Pregnant mothers with lower SRH and two or more comorbidities had a higher chance of having an abnormal level of anxiety subscale (6.9, 3.7 times, retrospectively).

**Conclusion:**

The results revealed that an abnormal level of depression was associated with SRH and medical insurance status. Moreover, the number of comorbidities and poor SRH significantly increased the chance of achieving abnormal anxiety levels in pregnant mothers during the COVID-19 pandemic.

## Plain English summary

Children of mothers who experience high psychological distress during pregnancy are more susceptible to cognitive and behavioral problems. Few studies have reported the psychological distress of pregnant mothers during the COVID-19 pandemic, and it may be considered as a risk factor for child developmental disorders.

In total, 540 out of 920 registered pregnant mothers in maternity clinics affiliated with Shiraz University of Medical Sciences answered the online questionnaire and were included in this study. An online self-administered data gathering tool was used so that the respondents felt more secure. The data gathering tool had three main parts: socio-demographic, obstetric and medical histories, and the short form of the Depression Anxiety Stress Scales (DASS-21) which consisting of 21 questions, 7 in each subscale; depression (DASS-D), anxiety (DASS-A) and stress (DASS-S).

Pregnant mothers who had two or more comorbidities or those with lower self-rated health (SRH) had a higher chance of having an abnormal anxiety level. Depression levels were higher in pregnant mothers who had no insurance. Additionally, depression symptoms were more prevalent in pregnant mothers who had low health status than in those with good or intermediated SRH.

In conclusion, the COVID-19 pandemic contributes to a significant increase in depression and anxiety symptoms among pregnant mothers. Moreover, lack of insurance, poor SRH, and having comorbidities are significantly associated with increasing depressive and anxiety scores.

## Background

In late December 2019, Chinese healthcare facilities in Wuhan, Hubei, China reported numerous pulmonary infection cases with an unknown type, named coronavirus disease 2019 (COVID-19) [1, 2]. The World Health Organization (WHO) declared this disease as a global pandemic on March 11, 2020 [3]. On January 24, 2021, more than 43 million cases with 999,000 deaths were confirmed in the United States of America, and around 98.2 million cases with 2,112,000 deaths were confirmed worldwide [4].

Recent studies have reported that patients with heart failure, cancer, elderly patients with underlying disease, and immunocompromised patients are frail and predominantly susceptible to severe outcomes associated with COVID-19 infection [5, 6]. Due to the pathophysiological and mechanical changes experienced during pregnancy, pregnant women and their fetuses could be a high-risk population and more prone to this infection too [7, 8].

Previous studies revealed the remarkable impacts of infectious disease outbreaks such as severe acute respiratory syndrome (SARS) on psychological distress, including depression and anxiety in pregnant mothers [9]. Moreover, the children of mothers who experience high psychological distress during pregnancy are more likely to have cognitive and behavioral problems and their communication skills are significantly affected [10, 11]. Despite the extensive effect of psychological distress on pregnant women and their children, there is a gap in our knowledge about these distress rates or levels. Additionally, few studies have reported psychological distress in pregnant mothers during the COVID-19 pandemic [12–14]. Hence, this study aimed to investigate the predicting factors of depression, anxiety, and stress levels of pregnant mothers in the initial stage of the COVID-19 infection in the southwest of Iran.

## Methods

### Study design and participants

This cross-sectional study was conducted between March 24 and April 7, 2020, in Shiraz, the fifth populous city of Iran, located in the southwest of the country. The study protocol was based on the Helsinki ethical principles for medical research and approved by the Ethics Committee of Shiraz University of Medical Sciences (SUMS) (IR.SUMS.REC.1398.1424).

The participants were pregnant mothers registered in maternity clinics affiliated with SUMS. Each maternity clinic secretary contacted mothers by phone and introduce the study and its goals to them. After the participants were reminded of their rights, they were asked to complete an online questionnaire. Moreover, at the beginning of the online survey, a text was written which had some parts. At first, we clarified the study's aim, the importance of the participants' honesty in answering the questions. Then we mentioned that the study was supported and approved by SUMS. Furthermore, we mentioned that the data would be used for the research aim, and the participants would have the option to complete the questionnaire or not. So, those who submitted the complete questionnaire were aware of all details mentioned above.

Pregnant mothers were excluded from the study if:

- They did not answer their phone three times.
- They were reluctant to participate.
- They had not been living in Shiraz for at least six months prior to the study.

**Fig. 1:**
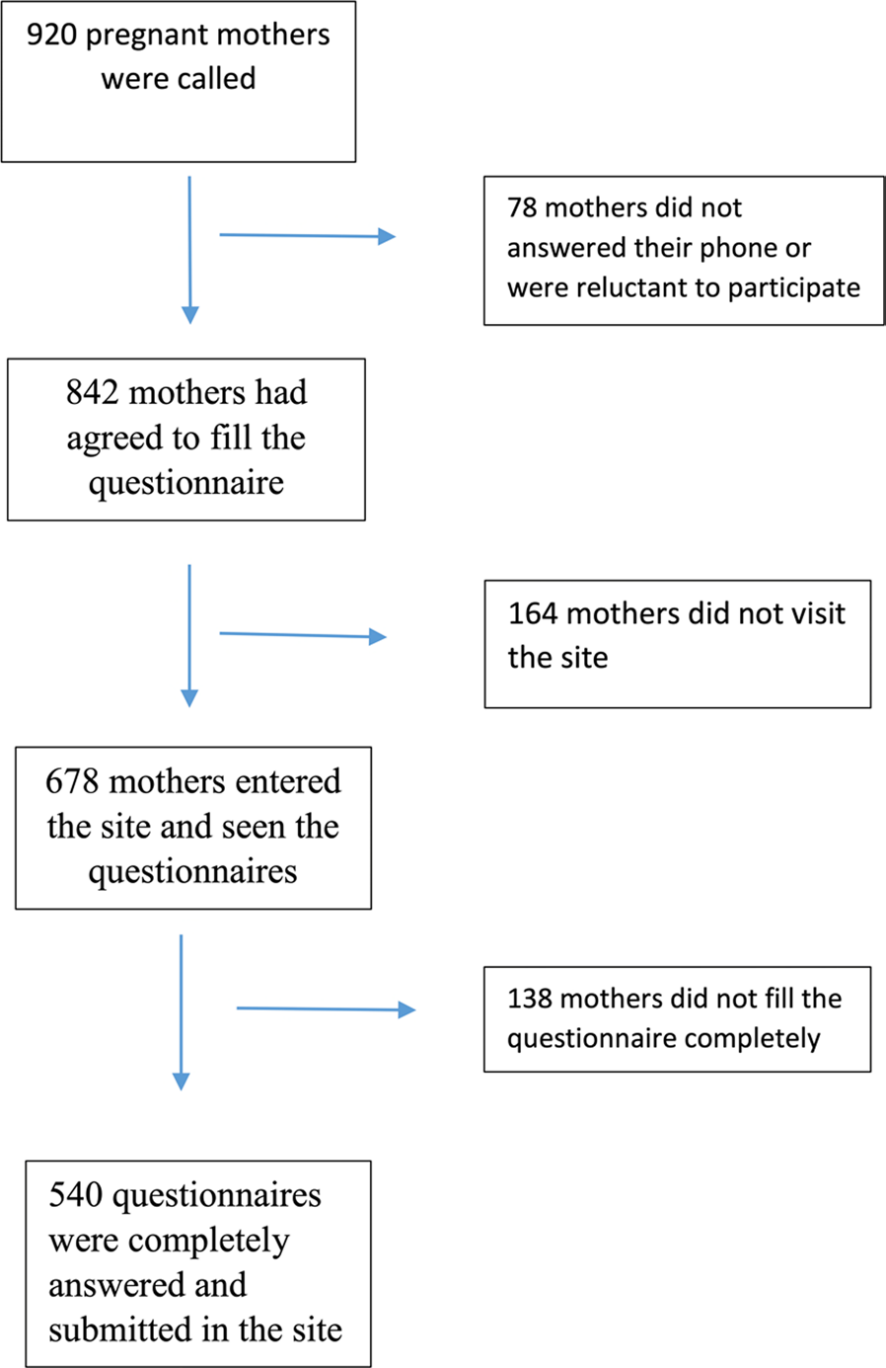
Flowchart of study

Ultimately, 540 pregnant mothers completed the questionnaire (Fig. 1).

### Data gathering tools

To assess the levels of depression, anxiety and stress in pregnant mothers, an online self-administered data gathering tool was used, so that the respondents felt more secure. The data gathering tool had three main parts: socio-demographic, obstetric and medical histories, and the short form of the Depression Anxiety Stress Scales (DASS-21).

The socio-demographic part queried the participant’s age, marriage duration, residential area (Shiraz versus villages around Shiraz), educational level, employment status, insurance status, self-reported socioeconomic status (SES), and the perceived correlation between the household income and expenditures.

The second part included gestational age (GA), number of pregnancies including the current one, concurrent maternal comorbidities, and medication history including the supplements.

The third part of the questionnaire comprised the DASS-21 questionnaire. The validity and reliability of DASS-21 was assessed by Asghari et al. in a sample of 378 non-clinical Iranian population. They showed that DASS-21 had an acceptable Cronbach’s alpha for the total score of DASS-21 (0.94) as well as for the subscales, including 0.85 for depression, 0.85 for anxiety, and 0.87 for stress [15].

The DASS-21 questionnaire consists of 21 questions, 7 in each subscale of depression (DASS-D), anxiety (DASS-A) and stress (DASS-S). Each question is scored on a 4-point Likert scale; 0 for “never”, 1 for “often”, 2 for “usually” and 3 for “always”. Hence, each subscale score ranges from 0 to 21, with higher scores representing the respondent’s higher levels of depression, anxiety, and stress. A participant was considered symptomatic (ranged from mild to severe) in the DASS-D, DASS-A, and DASS-S subscales if her scores were higher than 9, 7, and 14, respectively. The term “abnormal” was used for each DASS subscale if the participant achieved a score above the normal cutoff. Hence, an abnormal DASS conveyed that the participant was symptomatic, which could range from mild to severe symptoms.

### Statistical analysis

SPSS version 18.0 (IBM Corp., Armonk, NY, USA) was used to analysis the data. A Chi-square test was applied to assess the association between qualitative variables. Independent T-test and ANOVA were used to compare numeric variables between two groups and among three groups. In order to explore significant predicting factors of depression, anxiety and stress, first variables with *p*-values less than 0.2 in univariate analysis were entered into the logistic regression model and backward elimination (alpha-to-remove = 0.1) was used. A *P* value less than 0. 05 was considered statistically significant.

## Results

In total, 540 out of 920 registered pregnant mothers completed the online questionnaire (58.7%). The mean age of participants was 31.4 (± 5.9) years. The median duration of marriage was 7 years, ranging from 1 to 28 years. The median number of pregnancies, including the current one, was 2, ranging from 1 to 8 pregnancies (Table 1).

**Table 1:** Demographic characteristics of the participants

Variable	Mean (± SD)	Median (Min–Max)
Maternal age (year)	31.4 (5.9)	31 (17–49)
Marriage duration (year)	7.7 (5.3)	7 (1–28)
Number of pregnancies)	2.1 (1.1)	2 (1–8)

*SD* standard deviation, *Min* minimum, *Max* maximum

The majority of mothers had no comorbidity (451; 83.5%), while 59 mothers (10.9%) had one comorbidity, 29 mothers (5.4%) had two comorbidities, and one mother (0.2%) reported three comorbidities. While the first two most common comorbidities were hypothyroidism (42; 7.8%) and diabetes mellitus/gestational diabetes mellitus (31; 5.7%), the least frequent one was idiopathic thrombocytopenic purpura (ITP) reported by one pregnant mother (0.2%). It was found that 167 mothers (30.9%) had been taking medication; of them, 136 (25.1%) were taking supplements including ferrous sulfate, folic acid or perinatal multi-vitamins (Table 2).

**Table 2:** Medical histories of pregnant mothers

Comorbidities	Frequency (%)	Medication	Frequency (%)
Hypothyroidism	42 (7.8)	Ferrous sulfate	76 (14.1)
DM/GDM	31 (5.7)	Folic acid	59 (10.9)
Other endocrine disorders	20 (3.7)	Multi-vitamin	45 (8.3)
Headache	19 (3.5)	levothyroxine	42 (7.8)
HTN	17 (3.1)	Aspirin	42 (7.8)
CVD	9 (1.7)	Other	57 (10.6)
Renal diseases	9 (1.7)		
Respiratory diseases	7 (1.3)		
Seizure	7 (1.3)		
ITP	1 (0.2)		

*GDM* gestational diabetes mellitus, *HTN* hypertension, *CVD* cardiovascular diseases, *ITP* immune thrombocytopenic purpura

Table 3 shows the distribution of symptomatic participants in the DASS subscales based on the participants’ demographic and socio-economic factors. Having an above-normal DASS-D score was statistically associated with the respondent’s insurance status and self-rated health (SRH) level. Hence, an abnormal DASS-D score was significantly higher in pregnant mothers who had no insurance (10.5% vs. 3.9%; *p* = 0.01). An abnormal DASS-D score was more prevalent among pregnant mothers who reported poor health status (15.6%) compared with those who reported good SRH (6.1%) and intermediate SRH (3.1%). The proportion of abnormal DASS-D was evenly distributed among the remaining demographic and socio-economic factors.

**Table 3:** Comparing of abnormal DASS scores based on the pregnant mothers’ demographic and socio-economic status

Maternal information		Abnormal depression score	Abnormal anxiety score	Abnormal Stress score
	Frequency (%)	Frequency (%)	Frequency (%)	Frequency (%)
Maternal age (years)				
< 19 OR > 34 years	169 (31.3)	10 (5.9)	35 (20.7)	2 (1.2)
8 < age < 35 years	371 (68.7)	18 (4.9)	70 (18.9)	3 (0.8)
P value		0.4	0.3	0.5
Marriage duration (years)				
1–5 years	232 (43)	10 (4.3)	49 (21.1)	2 (0.9)
6–10 years	168 (31.1)	8 (4.8)	26 (15.5)	2 (1.2)
> 10 years	140 (25.9)	10 (7.1)	30 (21.4)	1 (0.7)
P value		0.5	0.3	0.9
Number of pregnancies				
First pregnancy	195 (36.2)	11 (5.6)	42 (21.5)	2 (1)
Second pregnancy	166 (30.7)	4 (2.4)	25 (15.1)	0
Third or more pregnancy	179 (33.1)	13 (7.3)	38 (21.2)	3 (1.7)
P value		0.1	0.2	0.2
Gestational age (weeks)				
< 14 weeks	36 (6.7)	3 (8.3)	7 (19.4)	0
4–28 weeks	184 (34.1)	5 (2.7)	41 (22.3)	1 (0.5)
> 28 weeks	320 (59.3)	20 (6.3)	57 (17.8)	4 (1.3)
P value		0.1	0.4	0.6
Residential area				
Urban	425 (78.7)	22 (5.2)	87 (20.5)	4 (0.9)
Rural	115 (21.3)	6 (5.2)	18 (15.7)	1 (0.9)
P value		0.6	0.1	0.7
Job status				
Housewife	488 (90.4)	25 (5.1)	88 (18)	5 (1)
Employed	52 (9.6)	3 (5.8)	17 (32.7)	0
P value		0.5	**0.01**	0.6
Highest educational attainment				
Below high school diploma	119 (22)	8 (6.7)	24 (20.2)	2 (1.7)
High school diploma	199 (36.9)	7 (3.5)	27 (13.6)	1 (0.5)
University degree	222 (41.1)	13 (5.9)	54 (24.3)	2 (0.9)
P value		0.4	**0.02**	0.6
Insurance status				
Insured	435 (80.6)	17 (3.9)	83 (19.1)	3 (0.7)
Uninsured	105 (19.4)	11 (10.5)	22 (21)	2 (1.9)
P value		**0.01**	0.4	0.2
Correlation between income and expenditure				
Equal	136 (25.1)	4 (2.9)	20 (14.7)	1 (0.7)
Expenditure > income	401 (74.3)	24 (6)	84 (20.9)	4 (1)
Income > expenditure	3 (0.6)	0	1 (33.3)	0
P value		0.3	0.2	0.9
Claimed socio-economic status				
High	42 (7.8)	2 (4.8)	9 (21.4)	0
Middle	256 (47.4)	13 (5.1)	47 (18.4)	2 (0.8)
Low	242 (44.8)	13 (5.4)	49 (20.2)	3 (1.2)
P value		0.9	0.8	0.7
Self-rated health				
Poor	122 (22.6)	19 (15.6)	46 (37.7)	2 (1.6)
Intermediate	257 (47.6)	8 (3.1)	45 (17.5)	3 (1.2)
Good	242 (44.8)	1 (6.1)	14 (8.7)	0
P value		** < 0.001**	** < 0.001**	0.3
Number of Comorbidities				
No comorbidity	452	22 (4.9)	76 (16.8)	3 (0.7)
1 comorbidity	58	4 (6.9)	15 (25.9)	1 (1.7)
> 1 comorbidity	30	2 (6.7)	14 (46.7)	1 (3.3)
P value		0.7	** < 0.001**	0.2

P values presented in bold are significant

P value < 0.05 was considered significant

Depression score > 9 was considered abnormal

Anxiety score > 7 was considered abnormal

Stress score > 14 was considered abnormal

DASS-A was significantly associated with pregnant mothers’ job status (*p* = 0.01), educational level (*p* = 0.02), SRH (*p* < 0.001), and number of comorbidities (*p* < 0.001). The findings indicated that abnormal DASS-A was more prevalent among employed mothers (32.7% vs. 18%), and those with a university degree (24.3%). Furthermore, mothers who reported their health status as poor had the highest frequency (37.7%) of abnormal DASS-A score compared with those reporting an intermediate SRH (3.1%) or good SRH (6.1%) groups. An abnormal DASS-A level was much more prevalent in respondents with 2 or more comorbidities than in those with one or no comorbidity; 46.7, 25.9, and 16.8%, respectively. No statistically significant differences were found in the distribution of abnormal DASS-A in the variable subgroups (Table 3).

DASS-S was evenly distributed among subgroups of all demographic and socio-economic factors, including maternal age, duration of marriage, number of pregnancies, gestational age, residential area, occupation, educational status, insurance status, income, claimed SES, SRH, and number of maternal co-morbidities (Table 3).

As demonstrated in Table 4, logistic regression analysis showed that an abnormal DASS-D was associated with SRH (*p* < 0.001) and insurance status (*p* = 0.03). Pregnant mothers who reported their health status as poor had a higher chance of having an abnormal DASS-D score (OR = 27.8; *p* = 0.001). Respondents who did not have insurance had a 2.5 times higher chance of having an abnormal DASS-D score (*p* = 0.03; CI for OR 1.1–5.6).

**Table 4:** Determinants of having an abnormal depression level according to the DASS scale

	Odd’s Ratio (OR)	95% Confidence Interval for OR	P value
Self-rated health			
Good	1		< 0.001
Intermediate	5.1	0.6–11.2	0.1
Poor	27.8	3.6–52.7	0.001
Having insurance			
Yes	1		0.03
No	2.5	1.1–5.6	

Depression score > 9 was considered abnormal

Table 5 shows those factors associated with an abnormal DASS-A level. According to the logistic regression, mothers with lower SRH had a higher chance of achieving a poor DASS-A score. Hence, those with poor SRH had a 6.9 times higher chance of having an abnormal DASS-A level (*p* < 0.001; CI for OR 3.5–13.7), while those who rated their health status as intermediate had a 2.3 times higher chance of achieving an abnormal DASS-A score (*p* = 0.01; CI for OR 1.2–4.4). Furthermore, number of comorbidities was associated with low DASS-A score; respondents who had two or more comorbidities had a 3.7 times higher chance of achieving an abnormal DASS-A level (*p* = 0.001; CI for OR 1.7–8.2).

**Table 5:** Determinants of having an abnormal anxiety level according to the DASS scale

	Odd’s Ratio (OR)	95% Confidence Interval for OR	P value
Self-rated health			
Good	1		< 0.001
Intermediate	2.3	1.2–4.4	0.01
Poor	6.9	3.5–13.7	< 0.001
Number of comorbidities			
No comorbidity	1		0.003
1 comorbidity	1.6	0.8–3.1	0.2
> 1 comorbidity	3.7	1.7–8.2	0.001

Anxiety score > 7 was considered abnormal

## Discussion

The COVID-19 pandemic may contribute to a significant increase in depressive and anxiety symptoms. The results of this study revealed that pregnant women repeated abnormal depression and anxiety levels in the initial stage of the COVID-19 pandemic. They further showed that pregnant mothers who had two or more comorbidities and those with lower SRH had a higher chance of having an abnormal anxiety level. A higher depression level was reported in pregnant mothers who had no insurance. Additionally, depression symptoms were more prevalent in pregnant mothers who had poor health status than in those with good or intermediate SRH. Pregnancy is a significant transition period in a woman’s life, and many physiologic and immunologic changes occur during this period [16]. Psychiatric disorders such as depression and anxiety during pregnancy have been associated with many complications, including preeclampsia, diabetes, premature birth, low birth weight, and postnatal complications [17].

Culture is a set of beliefs held by a group of people representing the way of life for that specific group. Cultural values and socioeconomic elements are firmly associated. Previous studies have confirmed that different cultural orientations play pivotal roles in social behavior such as aggression, anxiety, and depression. Moreover, in most developing countries, a high percentage of people are prone to severe stress caused by poverty, natural hazards, or violence. (Most people in high-income countries are protected against these misfortunes.) These factors have an astonishing effect on mental health. Several countries with different cultures are affected by depression, a common psychiatric disorder [18–20]. Changes in women's hormone levels may lead to an increased chance of depression progression twice that of men, especially during the reproductive period and pregnancy [21]. Previous studies reported a high rate of psychiatric morbidities such as depression and panic attack during the SARS outbreak in 2003. It may reflect another aspect of the importance of these infectious disease outbreaks and necessitate mental health evaluations during these periods [22, 23].

The current study showed that pregnant mothers who reported poor health status and those with no health insurance, had higher abnormal depression scores than others. In line with these results, Wang et al. reported that poor perceived health was highly associated with depression rates [24]. Ahorsu et al. reported the relationship between mental health and fear of COVID-19 among Iranian pregnant mothers and their husbands. Consistent with the current study, they found that fear of COVID-19 was significantly related to the depression level of Iranian pregnant women and their husbands [25].

Anxiety is another critical public health concern, because it can lead to impairments in social, emotional, and physical functioning, resulting in a higher level of healthcare service utilization [26]. Glover et al. revealed that increased anxiety during pregnancy had a significant relationship with plasma and amniotic cortisol levels [27]. Consistent with the current findings, Lebel et al. and Wang et al. recently reported that pregnant women had clinically elevated pregnancy-related anxiety symptoms during the COVID-19 pandemic [12, 24].

Conversely and inconsistent with the present study, Wu et al. and Durankus et al. reported that low education level was a common associated at-risk factor for depression progression and anxiety symptoms during the COVID-19 pandemic [17, 28]. The current results revealed that an abnormal anxiety level was more prevalent among pregnant mothers who had university degrees. This may be explained by the fact that pregnant mothers with a high level of education had more awareness about this pandemic's threat and consequences; thus, they are more affected mentally than low-educated pregnant mothers.

The current study had several limitations. First, this study was cross-sectional and we could not show the long-term effects of depression and anxiety levels on maternal and neonatal outcomes. Second, pregnancy is a physiologic condition accompanied by anxiety and stress due to hormonal changes as well as facing a new circumstance, and we could not differentiate the contribution of pregnancy and COVID-19 pandemic in higher levels of anxiety and stress reported by participants. However, former researchers have mentioned that these levels had been increased compared with the pre-COVID-19 pandemic era [12]. Moreover, the current study was conducted only in Shiraz, one of the largest cities in Iran, so the results cannot be generalized to the whole of Iran.

This study had some strengths as well. One of the strongest points of this study is that it was conducted during the first months of the epidemic. Therefore, it reflects the real stress and anxiety of expecting mothers. Moreover, this topic had not yet been addressed in Iran during the COVID-19 pandemic. Another positive aspect of this study is that women felt more comfortable about expressing their anxiety and depression symptoms on the self-administered questionnaire used herein.

## Conclusion

The current study results revealed that the COVID-19 pandemic may contribute to a significant increase in depression and anxiety symptoms among pregnant mothers. Moreover, a lack of insurance, poor SRH, and the existence of comorbidities are significantly associated with increasing depression and anxiety scores. Screening for psychological disorders such as depression and anxiety, especially in pregnant women, and well-known communication with consistent and precise updates about the COVID-19 pandemic should be provided as a disease-preventive plan for intellectual and cognitive well-being.

## Data Availability

Data supporting results in this article are filed and safely locked away in the office of the First Author (Dr. Najmeh Maharlouei) at the Shiraz University of Medical Sciences, Shiraz, Iran. The corresponding author is ready to avail of the said data at reasonable request.

## Abbreviations

COVID-19: Coronavirus Disease 2019
SARS: Severe acute respiratory syndrome
DASS-21: Depression Anxiety Stress Scales-21
SRH: Sexual and reproductive health
